# Exploring the Bile Stress Response of *Lactobacillus mucosae* LM1 through Exoproteome Analysis

**DOI:** 10.3390/molecules26185695

**Published:** 2021-09-20

**Authors:** Bernadette B. Bagon, Ju Kyoung Oh, Valerie Diane V. Valeriano, Edward Alain B. Pajarillo, Dae-Kyung Kang

**Affiliations:** Department of Animal Resources Science, Dankook University, Cheonan 31116, Korea; dette.bagon@gmail.com (B.B.B.); supajuko@gmail.com (J.K.O.); vdvaleriano@gmail.com (V.D.V.V.); eabpajarillo@gmail.com (E.A.B.P.)

**Keywords:** lactic acid bacteria, extracellular proteome, bile stress response, *Lactobacillus mucosae*, probiotics

## Abstract

*Lactobacillus* sp. have long been studied for their great potential in probiotic applications. Recently, proteomics analysis has become a useful tool for studies on potential lactobacilli probiotics. Specifically, proteomics has helped determine and describe the physiological changes that lactic acid bacteria undergo in specific conditions, especially in the host gut. In particular, the extracellular proteome, or exoproteome, of lactobacilli contains proteins specific to host– or environment–microbe interactions. Using gel-free, label-free ultra-high performance liquid chromatography tandem mass spectrometry, we explored the exoproteome of the probiotic candidate *Lactobacillus mucosae* LM1 subjected to bile treatment, to determine the proteins it may use against bile stress in the gut. Bile stress increased the size of the LM1 exoproteome, secreting ribosomal proteins (50S ribosomal protein L27 and L16) and metabolic proteins (lactate dehydrogenase, phosphoglycerate kinase, glyceraldehyde-3-phosphate dehydrogenase and pyruvate dehydrogenases, among others) that might have moonlighting functions in the LM1 bile stress response. Interestingly, membrane-associated proteins (transporters, peptidase, ligase and cell division protein ftsH) were among the key proteins whose secretion were induced by the LM1 bile stress response. These specific proteins from LM1 exoproteome will be useful in observing the proposed bile response mechanisms via in vitro experiments. Our data also reveal the possible beneficial effects of LM1 to the host gut.

## 1. Introduction

Beneficial microorganisms were introduced as probiotics more than one hundred years ago, and have been used widely since then, not only for prevention of food spoilage but also for improvement of nutrient absorption from non-digestible food and overall gut health [1]. Defined as, “*Live microorganisms that, when administered in adequate amounts, confer a health benefit on the host*”, a probiotic candidate must be identified genetically, must be able to survive the host gut environment, must provide health benefits to the host, must have information about the effects on various hosts, and must be tested adequately prior to probiotic application [2]. *Lactobacillus* sp. are good probiotic candidates due to their numerous existing applications in the food industry without adverse effects on consumers. Thus, lactobacilli have been granted GRAS (generally regarded as safe) status. In fermented dairy, meat and vegetables, as well as functional foods, lactobacilli have numerous current uses. In addition to their safety status and fermentation ability, lactobacilli have great potential for biotechnological application due to their ability to colonise diverse ecological niches [3]. Depending on the origin of a *Lactobacillus* isolate, favourable genetic characteristics such as survival and adaptation in the host gastrointestinal tract, regulatory or transport functions and interaction with the intestinal mucosa, can be observed. For example, because the host gut environment entails harsh conditions (e.g., low pH, bile acids), isolation of bacteria from this niche guarantees greater capacity to withstand this environment than other sources.

Proteomic studies have supported the use of lactobacilli as probiotics. Numerous proteome studies have elucidated specific proteins detected under different conditions (e.g., different media and in vitro environments), in response to a range of stressors (e.g., acid, ethanol, heat, bile, starvation, oxidation, osmosis), and even in food matrix systems [3,4,5,6]. These studies have revealed proteins with novel supplementary or moonlighting functions under such conditions [7]. Specifically, the subset of the proteome that can directly interact with the extracellular environment, called the exoproteome, has been of interest. In contrast to the surface proteome (surfaceome), which are proteins attached at the S layer of bacteria, the exoproteome (sometimes secretome) is made up of secreted proteins that naturally influence gut health and provide the first line of defence for bacterial survival [8,9].

In our previous investigations, we found that a natural inhabitant of the pig intestine, bacterial isolate *Lactobacillus mucosae* strain LM1, has probiotic characteristics including good adhesion ability, aggregation ability, pathogen inhibition ability and bile tolerance ability. Analysis of its genome and proteome have revealed specific genes and expressed proteins that support these abilities [10,11,12,13,14]. As of writing, we know that LM1 has strong carbohydrate-specific adhesion to intestinal epithelial cells (55% to 65%) and porcine mucin (70% to 85%). In turn, this carbohydrate-specific adhesion ability supported the LM1 ability of competitive displacement of pathogenic strains (22% against *E. coli* K88, and 10% against *S. enterica* ser. Typhimurium KCCM 40253) [10]. Proteomic analysis of LM1 interactions with the host intestine during co-culture with intestinal epithelial cells (IPEC-J2 cells) identified many proteins with protein synthesis functions (e.g., transcription, translation, ribosome synthesis) whose intracellular pathways were regulated by interactions with the host [11]. Interestingly, this group of proteins were also most abundant in the exoproteome of LM1 during normal conditions, when at mid-logarithmic growth phase, under little to no environmental stress [12]. These secreted intracellular proteins were then suggested to have moonlighting functions related to LM1 adhesion and aggregation abilities. In addition, investigations of the LM1 genome revealed many genes with benefits for gastrointestinal adaptation, such as glycogen metabolism, folate biosynthesis, and niche-adaptation genes [13]. It was also found that LM1 has bile salt hydrolase genes in its genome. In vitro, LM1 has demonstrated its ability to survive bile stress exposure (both 0.10% and 0.30% bile concentration) for 90 min [14]. Thus, we continue to investigate this ability of LM1 by examination of its exoproteome under bile stress via gel-free, label-free ultra-high-performance liquid chromatography (UHPLC) tandem mass spectrometry (MS/MS). This will further our knowledge of LM1 as a beneficial component in the gut and clarify its adaptations to that environment.

## 2. Results 

### 2.1. Overview of Lactobacillus mucosae LM1 Exoproteome during Bile Stress

**Figure 1:**
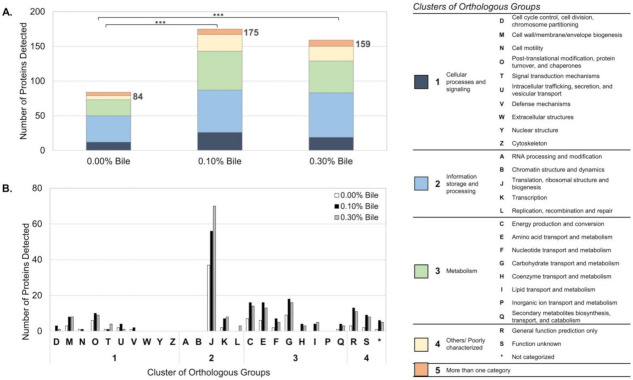
Composition and functional analysis of the *Lactobacillus mucosae* LM1 exoproteome under normal conditions (0.00% bile) and during bile treatment (0.10% and 0.30% bile), based on Clusters of Orthologous Groups, classified into (**A**) major and (**B**) specific functional categories. Tukey’s multiple comparisons test after ANOVA show protein secretion is significantly higher under bile treatment (*** *p* value < 0.001, *n* = 3). Specifically, proteins with COGs for [J] translation, ribosomal structure and biogenesis under [2] information storage and processing group of proteins were the most abundant.

**Figure 2:**
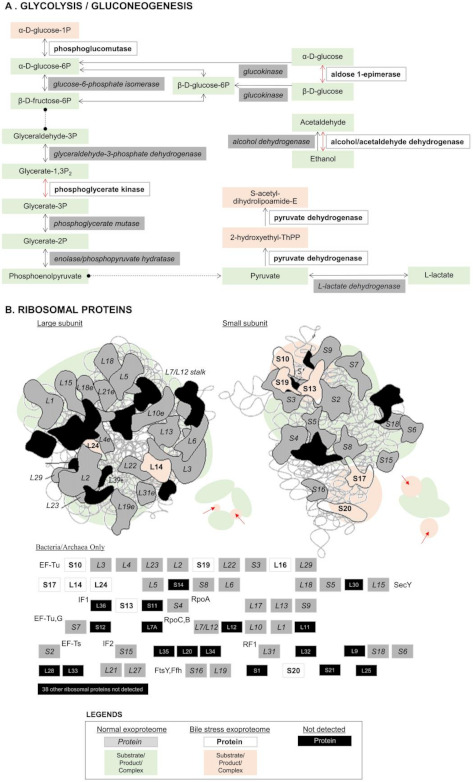
The top functional modules and pathways detected through KEGG-based analysis of the *Lactobacillus mucosae* LM1 exoproteome. The presence of specific proteins under normal conditions and bile stress (0.10% and 0.30% bile) mainly influenced the glycolysis/gluconeogenesis pathway (**A**) and specific components of the large and small ribosomal subunits (**B**).

**Figure 3:**
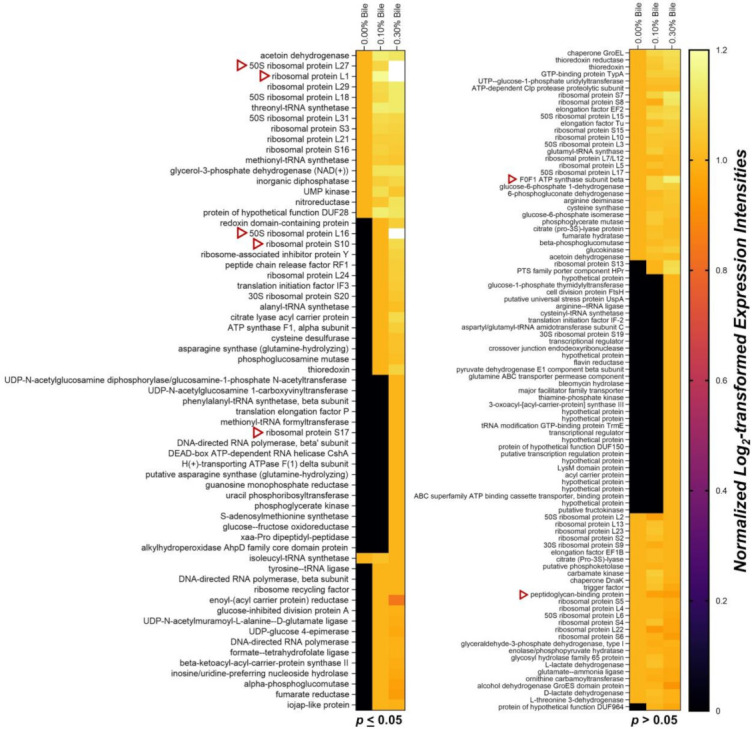
Abundance heatmap of the *Lactobacillus mucosae* LM1 exoproteome showing significant up or downregulation of secretion under bile stress (*p* < 0.05), as well as proteins with no statistically significant changes in abundance (*p* > 0.05). Red arrows indicate notable proteins with possible bile response roles.

The exoproteome of *L*. *mucosae* LM1 expressed in response to bile treatments was investigated in this study. Compared to the proteomes of other lactobacilli such as *Lactobacillus acidophilus* NCFM [15], *L. johnsonii* PF01 and CI-10 [12] and *L. rhamnosus* GG [16], LM1 has a large number of proteins in its exoproteome. A total of 228 unique proteins were identified, of which 144 were significantly influenced by bile (*p* < 0.05). Among many functions, proteins for translation, ribosomal structure and biogenesis were highly expressed extracellularly after bile treatment (Figure 1b, ‘J’). KEGG-based analysis also revealed a relevant presence of proteins from the large and small subunit of the ribosome (Figure 2). In particular, the abundance of the 50S ribosomal proteins L27, L1 and L16, which are involved in protein biosynthesis, were significantly upregulated (Figure 3). On the other hand, no significant changes were observed for some proteins already at high abundance under normal conditions (no bile), including phosphoketolase (xpk), glyceraldehyde-3-phosphate dehydrogenase (gapdh), cysteine synthase (cysK), enolase, molecular chaperone groEL, and many other ribosomal proteins (Figure 3).

### 2.2. Functional Annotation-Based Analysis of LM1 Exoproteome

The various functional annotation methods applied to the exoproteome revealed the adaptations of LM1 in response to bile stress. Based on COG analysis, numerous secreted proteins were predicted to have functions related to metabolism or information storage and processing (Figure 1a). In addition to the proteins for (J) translation, ribosomal structure and biogenesis, the proteins for (G) carbohydrate transport and metabolism, (E) amino acid transport and metabolism and (C) energy production and conversion were also highly elevated (Figure 1b). Moreover, proteins for (I) lipid transport and metabolism, (H) coenzyme transport and metabolism, and (D) cell cycle control, cell division and chromosome partitioning were secreted only when the cells were exposed to bile stress. 

Based on the predicted localization of the proteins, the LM1 exoproteome is composed primarily of cytoplasmic or intracellular proteins, with few secreted or extracellular proteins (Table 1). Proteins associated to the cell wall or anchored to the membrane were secreted in the presence of bile. These proteins include ABC superfamily ATP-binding cassette transporter (binding protein, hisJ), peptidase U34 dipeptidase (pepD), UDP-*N*-acetylmuramoyl-l-alanine-d-glutamate ligase (murD), cell division protein FtsH (hflB), glutamine ABC transporter permease component (hisM) and major facilitator family transporter (melB).

In KEGG-based functional annotation of the LM1 exoproteome, specific modules or pathways were identified that may be influenced by bile stress (Figure 2). Most notable among these are the glycolysis/gluconeogenesis pathway and components of the large and small subunits of the ribosome. Here, we note reactions that may occur extracellularly under normal and bile stress conditions. Among key proteins that may be responsible for the bile response are phosphoglucomutase (pgm), phosphoglycerate kinase (pgk), aldose-1-epimerase (galM), alcohol/acetaldehyde dehydrogenase (adhE) and pyruvate dehydrogenase (pdhA and pdhB). Ribosomal proteins from the 50S large subunit (L14, L16, L24) and 30S small subunit (S10, S13, S20, S17, S19) were also identified.

### 2.3. Intensity-Based Abundance Analysis of the LM1 Exoproteome

Heatmap analysis based on an abundance of the proteins under normal conditions (no bile) revealed bile-induced upregulation of 50S ribosomal proteins L27 and L1 secretion, along with 14 other proteins (Figure 3, *p* ≤ 0.05). Significant downregulation of secretion was observed only for isoleucyl-tRNA synthetase (ileS).

Heatmap analysis also indicated significant secretion of new proteins at different bile concentrations. Figure 3 shows that 50S ribosomal proteins L16 and 28 other proteins were secreted after treatment with 0.10% bile. Then, 0.30% bile influenced the secretion of those proteins and also induced the secretion of phosphoglycerate kinase and 16 other proteins.

Interestingly, relatively consistent secretion of 58 proteins in the normal exoproteome was observed under bile stress (Figure 3, *p* > 0.05). During bile treatment, F_0_F_1_ ATP synthase subunit beta (atpD) exhibited upregulation and peptidoglycan-binding protein (lytE) was downregulated. The abundance of these proteins changed, but the differences were not significant. Lastly, secretion of 37 other proteins was induced by 0.10% and 0.30% bile (*p* > 0.05 based on LFQ intensities).

## 3. Discussion

### 3.1. Bile-Induced Changes in the LM1 Exoproteome

We prepared cultures at the mid-logarithmic growth phase to minimize responses induced by other stress factors (such as low nutrients, low pH) during the stationary phase [12,17,18,19]. The known ability of LM1 to survive up to 0.30% bile [14] tells us it must be utilizing some proteins to help it survive the stressful environment. From the large exoproteome size of LM1 during normal conditions [12], we find that LM1 secretes a lot of cytoplasmic proteins naturally. In the current study, we observed bile-induced changes in the composition of the LM1 exoproteome (Figure 1). The significant difference in exoproteome composition (size and identities) between two bile treatments (0.10% and 0.30% bile) suggests specific sets of proteins (mostly intracellular proteins) are being secreted by LM1 for its response. Instead of cell lysis, the current exoproteome data suggest that LM1 modifies its exoproteome by secreting intracellular proteins as a response to bile stress.

Under normal conditions, without bile, proteins for both information storage and processing and those with roles in metabolism are abundant (Figure 1a). Although proteins of all functions increased with bile treatment, the dominant functions remained information storage and processing (translation, ribosomal structure and biogenesis) and metabolism (carbohydrate, amino acids, lipid, coenzymes and energy conversion). Cooperation between proteins related to translation and metabolism is a possible explanation of this pattern. A similar tendency was previously observed in our analysis of two strains of *L. johnsonii,* which adapted to bile stress by optimising protein synthesis while maintaining their supply of energy [20]. The specific modules and pathways detected through KEGG-based analysis, glycolysis/gluconeogenesis and ribosomal proteins, support this functional adaptation in LM1. Metabolic enzymes including pgm, pgk, galM, adhE, pdhA and pdhB, may have been secreted to boost the energy production of LM1 by completing existing metabolic pathways, while additional ribosomal protein components could help optimize protein translation under stress (Figure 2).

However, as *L. mucosae* is not typically included in the *acidophilus* complex of lactic acid bacteria with *L. johnsonii,* differing extracellular processes may occur in these two species. Previously, we revealed that the LM1 exoproteome under normal conditions (without bile) could contribute to its aggregation and adhesion abilities, especially cytoplasmic proteins with moonlighting functions, such as gapdh, groEL and elongation factor Tu (ef-Tu) [12]. These same secreted proteins with moonlighting auto-aggregation functions may be able to protect LM1 during exposure to bile.

### 3.2. Bile Stress-Response Proteins in the LM1 Exoproteome

Analysis of localization and abundance intensities narrowed the specific bile stress-response proteins of LM1 to cell wall or membrane anchored proteins and a few ribosomal proteins. As *L*. *mucosae* LM1 secretes both intracellular and extracellular proteins with metabolic or translational functions, even without bile stress, the small number of cell wall and membrane anchored proteins detected only under bile treatment suggests specific functions in the bile response (Table 1).

At low bile concentration (0.10%), the membrane-associated proteins hisJ, pepD and murD were secreted. Based on the results of bioinformatics analysis, the ABC-type transporter hisJ is predicted to be a periplasmic N-terminally anchored membrane protein. Meanwhile, the peptidase pepD and ligase murD are cytoplasmic N-terminally anchored membrane proteins. All three proteins may be secreted via the general Sec translocase/signal peptidase I (Sec/SPI) pathway. Although Gram-positive bacteria such as LM1 lack the large periplasmic region of Gram-negative bacteria, the established roles of these three proteins suggest collaboration in the transport of bile out of the cell, breakdown of essential protein sources and simultaneous biosynthesis of the cell wall. An efflux-based mechanism for bile acid resistance has also been proposed for an ABC-type transporter in *Lactococcus lactis* [21]. On the other hand, previous studies of the bile stress response of *Lactobacillus casei* Zhang and *Bifidobacterium animalis* subsp. *lactis* showed that peptidases and ligases were downregulated intracellularly [22,23]. Thus, extracellular bile response roles of the two membrane-associated proteins are first proposed for LM1. With 0.30% bile treatment, three new membrane-associated proteins were secreted, namely the cell division protein ftsH (or hflB), and the transporters hisM and melB. These three multi-transmembrane proteins are secreted via the Sec/SPI pathway. This high bile concentration may have demanded upregulation of proteins responsible for the transport of bile out of the cell. In addition, ftsH is involved in bile stress tolerance in *Lactobacillus plantarum* WCFS1, with a specific role in protein quality control, and supports greater survival of bacteria [24]. Thus, it may also increase LM1 survival under high levels of bile stress.

Interestingly, many ribosomal proteins exhibit bile-induced secretion. A similar observation by [16] in the *L. rhamnosus* GG surface-exposed proteome revealed that ribosomal protein levels increase after bile stress. As ribosomal proteins are generally cytoplasmic, these surface-exposed ribosomal proteins were suggested to be anchorless surface proteins with possible immunomodulatory roles. This finding is in accordance with LM1 not undergoing cell lysis when releasing these cytoplasmic ribosomal proteins. Among the ten ribosomal proteins revealed in our results (heatmap and KEGG-based analysis), the 50S ribosomal proteins L27 and L16 appear to play key roles, as L27 secretion was strongly upregulated and L16 secretion was highly induced. In *Escherichia coli,* L27 plays a key role in the assembly of all 50S ribosomal subunits [25]. In addition, an *E. coli* mutant lacking L16, among other proteins, exhibited an assembly bottleneck, creating only the 40S ribosomal precursor. As proposed in our previous study of the *L. johnsonii* exoproteome [20], these bacteria may optimise their protein synthesis in harsh environments. Detection of these two proteins extracellularly or under bile stress has not been reported in other bacteria to date.

### 3.3. Consistent Abundance of Proteins in the LM1 Exoproteome under Bile Stress

Although we observed bile-induced changes in LM1 exoproteome composition, many of the proteins detected were also secreted under normal conditions, without bile. The consistent secretion of these proteins represents the normal extracellular function of LM1 and suggests that no damage to cell integrity occurred under bile stress.

The top 20 proteins in the LM1 exoproteome reported in a previous comparative analysis [12] were revisited in this study. All 20 proteins, including xpk, gapdh, cysK, enolase and groEL, were found to lack significant changes in abundance under bile stress. This finding demonstrates that LM1 consistently utilises proteins in the phosphoketolase pathway (PKP) in response to bile stress. The moonlighting functions of gapdh, groEL and ef-Tu under normal conditions (no bile) for LM1 cell adhesion were also unaffected by bile stress. The presence of many ribosomal proteins under both stress and normal conditions provides further evidence of sustained functionality in LM1 under bile stress.

Although not statistically significant, atpD showed the greatest amount of upregulation and lytE exhibited the strongest downregulation among the investigated subset of proteins (Figure 3, *p* > 0.05). F_0_F_1_-ATPases, including atpD, are generally upregulated in response to acid stress [3]. In terms of bile stress, atpD and other ATPases are downregulated or have low abundance [26]. However, as previous studies involved the intracellular proteome of lactobacilli, our finding provides a novel perspective on the exoproteome of lactobacilli. Interestingly, among all F_0_F_1_-ATPases, only atpD was detected in the LM1 exoproteome, suggesting that it has moonlighting functions unrelated to the acid stress response. On the other hand, lytE is a minor autolysin with lysM domains that binds to peptidoglycan. LytE is normally expressed during the logarithmic growth phase in *Bacillus* sp. and is functionally related to cell division [27]. Although it has not yet been fully explored in lactobacilli, the reduced presence of lytE in the exoproteome of LM1 under bile stress provides new insights into its regulated secretion and function in the bile stress response.

### 3.4. Possible Secretion Mechanisms of Intracellular Proteins Moonlighting Extracellularly

Similar to our study on *L. johnsonii* exoproteome under bile stress [20], we were mostly able to discuss LM1 exoproteome in the perspective of its intracellular functions. It is not clear if these proteins function the same extracellularly but it can be a possibility, in the same way as *L. johnsonii* uses its metabolic proteins outside. Since these proteins are evidently secreted extracellularly by LM1 when subjected to bile treatment, their moonlighting function as bile response proteins is definitely suggested. However, the lack of support from the predicted localization and secretion pathways for most proteins demands further studies to propose a possible mechanism behind their secretion.

Since we know LM1 produces a large exoproteome, even under normal conditions, and the abundance of these proteins do not significantly change under bile stress, cell lysis may not have contributed to the presence of both new and existing proteins. Excluding that possibility, post-translational modifications could be a mechanism that would allow the proteins to be secreted. Other than that, it is also possible that these proteins have conserved or specific domains that signal secretion with or without stress, but have not yet been studied intensively [20]. More experiments will be needed to confirm these mechanisms in LM1.

### 3.5. Host-Specific Beneficial Proteins in the LM1 Exoproteome

Identification of the proteins introduced to the extracellular environment by LM1 when subjected to a stressor present in the gut, specifically bile, allowed this study to investigate LM1 as a potential probiotic. Many proteins that are beneficial to the host or other microbes were secreted extracellularly by LM1. For example, the presence of Ldh and the metabolic proteins listed above (such as gapdh, enolase, pgk, pdhA and pdhB) offers a clear view of its contribution to host energy production in the gut (via glycolysis and lactic acid fermentation). In *L. plantarum,* the 30S ribosomal proteins S10 and S17 were upregulated during fermentation, suggesting functions in metabolism [28]. These two ribosomal proteins were also found in the exoproteome of LM1, where they may play the same metabolic role, as may the other ribosomal proteins detected (including L27, L1, L16, L14, L24, S13, S20 and S19). In addition, the moonlighting proteins that contribute to its aggregation and adhesion abilities (such as gapdh, GroEL and ef-Tu) allow LM1 to competitively exclude pathogens from the gastrointestinal tract, such as *E. coli* and *Salmonella enterica* Typhimurium [10,12,14]. Some of these extracellular proteins can also interact with other beneficial microbes and enhance their adhesion abilities, similar to the gapdh-like protein from the surface of *L. plantarum* WCFS1 [29]. This protein not only supports the adhesion of WCFS1 but also improves the adhesion of other lactobacilli to human epithelial cells (HT-29 cells). Lastly, potential immunomodulatory roles related to these extracellular ribosomal proteins (as observed in *L. rhamnosus* GG) will benefit host health.

## 4. Materials and Methods

### 4.1. LM1 Growth Conditions, Bile Treatment and Protein Collection

*Lactobacillus mucosae* LM1 isolated from porcine intestine was cultured in Difco de Man–Rogosa–Sharpe broth (BD Biosciences, San Jose, CA, USA) at 37 °C under static conditions. Seed cultures were prepared in 100 mL broth and incubated for 24 h.

Bile treatment followed a modified version of the procedures reported by [16]. Cultures were inoculated in 500 mL broth (1%) and incubated at 37 °C. To simulate human bile with similar ratio of glycine-conjugated to taurine-conjugated bile acids [30], Ox gall (SD Biosciences, Ontario, Canada) or bovine bile powder (B3883, Sigma-Aldrich, St. louis, MO, USA) were used. Ox gall or bovine bile solutions (0.0%, 0.1% and 0.3%; *v*/*v*) were added once the cultures had reached mid-logarithmic growth phase (optical density at 600 nm, 0.7 to 0.8). The bile-treated cultures were then incubated at 37 °C for 1.5 h. Three independent replicates were prepared.

Supernatants were harvested through centrifugation (8000 rpm, 10 min, 4 °C), filtered through a cellulose acetate membrane (0.22 μm pore size, 0.5 atm vacuum) and saturated to 80% via ammonium sulphate precipitation (4 °C, 12 h). The concentrates were centrifuged (3000× *g*, 20 min, 4 °C), and the resulting precipitates were dissolved in sodium citrate buffer (20 mM, pH 5.0) prior to dialysis using a regenerated cellulose dialysis membrane (Standard Grade, Spectra/Por; 1 kD cut-off) and 25 mM phosphate buffer (pH 7.0, 4 °C). During dialysis, the buffer was replaced twice at 2 h intervals, and then the protein was left to equilibrate at 4 °C (12 h). Proteins were separated through sodium dodecyl sulphate-polyacrylamide gel electrophoresis (12% resolving gel) and their concentration was determined with the Bradford protein assay. In-solution digestion was then performed as described previously [31].

### 4.2. Mass Spectrometry Analysis of LM1 Extracellular Proteins

Mass spectrometry was performed as described previously by [12]. Briefly, tryptic digests were separated through reversed-phase chromatography. Then, the peptides were separated using a UHPLC instrument (Dionex Ulti-Mate^®^ 3000; Thermo Fisher Scientific, Waltham, MA, USA) with Acclaim PepMap 100 trap and capillary columns. Specifically, fractions were reconstituted and resolved using solvent A (water/acetonitrile, 98:2 *v*/*v*; 0.1% formic acid) prior to running a liquid chromatography analytical gradient with increasing concentrations of solvent B (0.1% formic acid in acetonitrile).

Mass spectra were generated via coupling of the UHPLC instrument to a heated electrospray ionisation source (HESI-II) and the quadrupole-based Q Exactive™ Orbitrap High-Resolution Mass Spectrometer (MS; Thermo Fisher Scientific). The peptides obtained from UHPLC were electrosprayed through a coated silica tip (PicoTip emitter, New Objective, Littleton, MA, USA) (2000 eV ion spray voltage), and MS spectra were obtained at a resolution of 70,000 (200 *m*/*z*) over the 350–1800 *m*/*z* mass range, with a maximum injection time of 100 ms for ion accumulation. Eluted samples were used for successive MS/MS analyses. Ion activation and dissociation was performed using the high mass accuracy Orbitrap with higher-energy C-trap dissociation at a collision energy of 27 in the mass range of 100–1650 *m*/*z*.

The MaxQuant search engine was used to cross reference the raw quantified proteins to the proteome database available from UniProt [32]. The proteins of *L. mucosae* LM1 were annotated through matching of the sequences obtained in this study to the predicted proteome data in UniProt (UP000003645).

### 4.3. Bioinformatics and Statistical Analysis of the LM1 Exoproteome

The identified proteins were functionally annotated using the Clusters of Orthologous Groups (COG) method from the complete genome (GenBank GCA_000248095.3). In addition, LocateP v2.0 [33] and SignalP 4.0 [34] were used with the default settings and cut-off values to determine the localization and secretion pathways of the proteins. Proteins were also annotated using Kyoto Encyclopedia of Genes and Genomes Orthology (KEGG; KO) via the BlastKOALA tool [35] to investigate possible biological modules or pathways affected by the exoproteomes.

Statistical analysis was performed following the methods reported in a previous study [20]. Prior to further analysis, two-way analysis of variance (ANOVA) was performed using raw label-free quantification (LFQ) intensity data of three biological replicates of LM1 per group (0.00%, 0.10% and 0.30% bile). Statistically significant variance of protein abundance (*p* < 0.0001) for column factors (bile treatments) and row factors (each individual protein detected) was found, thus supporting the continuation of the comparative analysis. All LM1 protein abundance based on LFQ intensities were log_2_ transformed and normalised. For comparative analysis, proteins not expressed in a specific treatment were assigned values of zero prior to statistical analysis. Two-way ANOVA (significance at *p* < 0.05) was then performed, followed by Tukey’s multiple comparison test for post hoc analysis (GraphPad Prism 8.4.2, GraphPad Software, San Diego, CA, USA), to identify significant abundance changes during bile treatment [36]. A heatmap of protein abundance was constructed using the normalised log_2_-transformed values and divided based on statistical significance.

## 5. Conclusions

In this investigation of the potential probiotic application of *Lactobacillus mucosae* LM1, we explored its exoproteome under bile stress and interpreted the response of its protein composition from multiple perspectives. LM1 produces a large exoproteome in the absence of stress. With the introduction of bile (0.10% and 0.30%), LM1 adapted its exoproteome by increasing the levels of existing proteins as well as secreting new proteins. The change in exoproteome composition could lead to activation of the moonlighting functions of LM1 metabolic proteins (pgm, pgk, galM, adhE, pdhA and pdhB) rather than increasing metabolism or optimising protein synthesis. In addition, membrane-associated proteins (ftsH, pepD, murD, HisJ, HisM and MelB) and ribosomal proteins (L27, L1, L16, L14, L24, S10, S13, S20, S17 and S19) play direct roles in the LM1 bile stress response. These proteins may perform their primary functions and moonlighting activities simultaneously to increase LM1 survival. Lastly, the LM1 exoproteome contains many proteins that are functional in the gut and beneficial to the host with roles such as metabolism, adhesion and pathogen exclusion. This result suggests that LM1 and its exoproteome contribute to maintaining homeostasis in the host gut. As next steps, the putative bile response proteins of LM1 will be designed for specific mechanism confirmation experiments.

## Figures and Tables

**Table 1:** Cell wall and membrane-associated proteins with possible bile response roles, extracted from *Lactobacillus mucosae* LM1 exoproteome after determination of cellular localizations of all 228 proteins using LocateP v2.0.

Predicted Localization		Number of Proteins			Cell Wall and Membrane-Associated Proteins		
		0.00% Bile	0.10% Bile	0.30% Bile	0.00% Bile	0.10% Bile	0.30% Bile
**Cellular Destination**	Cytoplasmic	79	91	44	-	-	-
	Membrane	0	2	4	-	LBLM1_16160 MurD, LBLM1_00750 PepD	LBLM1_19820 HflB, LBLM1_21140 HisM, LBLM1_08510 MelB, LBLM1_16160 MurD *
	Cell Wall	1	0	0	LBLM1_10850 hypothetical protein EmrA	-	-
	Extracellular	4	0	3	-	-	-
**Subcellular Localization**	Intracellular	79	91	44	-	-	-
	Multi-transmembrane	0	0	3	-	-	LBLM1_19820 HflB, LBLM1_21140 HisM, LBLM1_08510 MelB
	LPXTG Cell wall anchored	1	0	0	LBLM1_10850 hypothetical protein EmrA	-	-
	N-terminally anchored	0	2	1	-	LBLM1_16160 MurD, LBLM1_00750 PepD	LBLM1_16160 MurD *
	Lipid-anchored	0	0	1	-	-	LBLM1_04250 hypothetical protein
	Secretory	4	0	2	-	-	-
**Localization Class**	Cytoplasm	76	84	41	-	-	-
	Inner membrane	0	4	3	-	LBLM1_15150 AbiF, LBLM1_04880 GlnQ, LBLM1_10270 GlnQ, LBLM1_21130 GlnQ	LBLM1_19820 HflB, LBLM1_21140 HisM, LBLM1_08510 MelB
	Periplasm	2	3	4	-	-	-
	Secreted	6	2	3	-	-	-

* Normalized log_2_-transformed intensity of this protein decreased during 0.30% bile treatment.

## Data Availability

The data presented in this study are available in the LM1 Bile Exoproteome (raw) Supplementary Materials.

## Supplementary Materials

The following are available online, SupplementaryFile_LM1BileExoproteome.xlsx.
